# On Ocular Symptoms in Tabes Dorsalis

**Published:** 1916-06-10

**Authors:** 


### ON OCULAR SYMPTOMS IN TABES DORSALIS.

### Local Symptoms of a General Import.

The recognition of tabes dorsalis or locomotor ataxia is not infrequently a very simple diagnostic achievement. When a story of pains of a shooting character is associated with an ataxic gait, with Romberg's sign, and with absence of the tendon jerks, a ready and confident conclusion is at hand. This may be called the typical picture of the disease, and it is here that the justification for the name "locomotor ataxia" is found. Since the recognition by Argyll Robertson of the peculiar character of the pupils the pupillary condition has been widely recognised and is now well established as valuable evidence in the diagnosis. Small pupils which do not respond to light, but which contract in the act of convergence, may in rare instances be found apart from locomotor ataxia, but in the great majority of instances their meaning is equivalent to this term. From this observation it has happened that more attention has been paid to eye conditions generally in the study of organic nervous diseases, and much helpful information has in this way been obtained. Indeed, nowadays, it must be considered a very defective examination of the nervous system which does not include a systematic investigation of the whole of the apparatus of vision. In this fashion it has become gradually established that the ocular disturbances in tabes dorsalis are not limited to the type of pupil described by Argyll Robertson, but that two other conditions must be added—namely, ocular palsies and optic nerve atrophy. Further, while such events may appear with other evidences of the disease and may thus merely confirm what the character of the gait and the defect of the tendon jerks establish, it is quite possible that one or more of these ocular symptoms may be the first clinical evidence of the disease. This is not all. It remains to be said that such a condition may long precede any signs of tabes dorsalis in its more characteristic form, and thus on the practical plane tabes dorsalis may for months, or years even, be displayed purely in the form of ocular and visual disturbances. This consideration alone is sufficient to show that conditions which seem to the patient to be concerned solely with his power of sight may well be the index or beginning of widespread disease. Hence the great importance of looking at such conditions in their true perspective and not allowing them to be matters of local concern and so-called special practice. They demand, each one of them, a complete and often a repeated examination of the whole of the nervous apparatus, and indeed of the body generally. Only in this way can a wise diagnostic conclusion be reached and the interests of the patient be protected.

Granted the above general conclusions, it follows that a patient who shows either optic atrophy, or an ocular paralysis, or Argyll Robertson pupils must be considered as a possible subject of tabes dorsalis, unless evidence to support an alternative diagnosis is forthcoming. There may be no disturbance of gait, no lightning pains, and no alteration of the tendon jerks. In spite of this the suspicion holds good, and, of course, it will be definitely strengthened if the patient has reached an age when degenerative nervous disease is not unlikely, or if he includes in his history an account of specific infection. Even in early life it must be remembered that the possibility of specific taint has to be reckoned with, and it has more than once been found that an ocular palsy in the early years of life has been the first step in the evolution of a juvenile tabes dorsalis. Of course, these statements do not involve the proposition that every case of ocular palsy unaccompanied by other evidence of organic nervous disease is an example of tabes dorsalis. Fortunately, experience confidently negatives such a conclusion. There are many such cases in which a paralysis of an ocular muscle is due to nothing more serious than a neuritis of the nerve which supplies it, and such neuritis appears often to be consequent on exposure to cold or chill. But, especially when it occurs in middle life and in a patient who has suffered from specific disease, such a paralysis raises the possibility of a wider diagnosis, and though in individual instances there may be circumstances which exclude this, there are many cases in which time alone will resolve the doubt. Thus, in brief phrase, it has to be said that an ocular palsy, even though it is of brief duration, may be the first sign of a commencing tabes dorsalis, and in calculating the future this possibility has to be borne in mind.

In regard to optic nerve atrophy a similar position has to be stated, but with greater emphasis of suspicion. Such relatively comfortable explanations as "chill" or "rheumatism" may hold good for an ocular paralysis, but they will not satisfy the position created by optic atrophy. Here there is certainly serious mischief afoot, and the outlook for the patient's sight is an anxious one, even when all other signs of organic nervous disease are wanting. On the other hand, it is fairly well established that in not a few cases where optic atrophy is the first step of tabes dorsalis the spinal evidences of the disease may be postponed for many years, and thus it may be said that the general prognosis is relatively good. Of course there may be evidences of nervous disease other than tabes dorsalis, as, for example, disseminated sclerosis, and here it is rare for the atrophy to proceed to complete blindness. But excluding this possibility, and excluding also cases of poisoning by tobacco or other toxic agent, bilateral optic atrophy as an isolated event brings the patient into the tabes dorsalis category. As a practical point it ought to be remembered that atrophy is quite consistent with good central vision, at least for a time, and that to detect its beginnings it is necessary to trace the extent of the visual field by means of the perimeter —a further illustration of the impossibility of drawing a sharp line of distinction between local ocular conditions and general nervous disease.

Whether, like ocular palsies and optic atrophy, the Argyll Robertson pupil may be the first of the symptoms of tabes dorsalis is a difficult question to answer with confidence. The disturbances of sight due to the two latter bring the patient promptly to his doctor, but an Argyll Robertson pupil inflicts no appreciable disability. Hence it is apt to pass unperceived unless it is associated with other symptoms. But judging from analogy it is likely to occupy in tabes dorsalis a similar position to that occupied by an ocular palsy or an optic atrophy, and its recognition ought to be followed by the same conclusions which these two conditions impose.

